# A Case of Trance

**Published:** 1891-09

**Authors:** 


### A CASE OF TRANCE.

Miss Cora Mattoon, of Plymouth Centre, Ct., was attacked with the grip on May 6, and a few hours later sank into a trance, from which she did not recover consciousness until May 20. Dr. Peas, of Thomaston, was the physician in charge of the case, and he administered nourishment as best he could to sustain life. When Miss Mattoon recovered, the first person she recognized was her sister, and she greeted her with the strange question : Are you dead, too?

The family thought the young lady's mind was wandering, but in a short time, and as soon as she gathered strength enough to talk, they were told a strange story, which, as the young lady is perfectly sane, is considered the queerest experience ever heard. It can best be told in her own words, as related to a *News* man, in an interview to-day : When I became unconscious, said Miss Mattoon, I saw strange and new surroundings, as plain as I do the furniture of this room. I thought I was dead, and I could see them placing me in a large vault. Then I met two cousins whom I know, but who are both dead, and a deceased uncle whom I never saw before. They told me I was dead, but when I inquired about my future state, they would give me no reply. They took me from the vault to the top of a mountain. There I saw many strange animals, but they were gifted with speech, and conversed among themselves and with me. I was left alone on the mountain, from the top of which I saw many beautiful scenes, and some horrible ones that I cannot describe. After that I went to the ocean and descended beneath the waves to the bottom. Slimy fish and terrible serpents crawled around me in great numbers. I remained at the bottom of the sea for some time, and then returned to Plymouth, where I met on the streets former residents whom I never saw, and who died years ago. They talked with me and told me I was dead, but when I asked them the same question I had addressed to my uncle and cousins, as to the nature of my future home, they would tell me nothing. While in Plymouth I met Jennie Hawkins, a girl friend of mine who was alive and well when I was taken sick. She said to me : I am dead, and you are dead, too. I really believed that I had passed out of life, and when I awoke from the trance, and saw my sister, I imagined she was dead also. It was several days before I could collect my thoughts and feel as though I was alive and on earth.

Miss Mattoon's story has created the most profound sensation. The strangest portion of it is, Jennie Hawkins, the friend she met while in the trance, and who told her she was dead, really did die during Miss Mattoon's illness. There is all manner of speculation in regard to the case, but no one has offered a satisfactory explanation.

Miss Mattoon is a bright, intelligent girl, and is a member of one of the best families in Plymouth Centre. Her veracity is undoubted, and the only verdict is that she has had a very remarkable experience. —*Progressive Thinker.*

